# Testing early life effects frameworks: developmental constraints and adaptive response hypotheses do not explain fertility outcomes in wild female baboons

**DOI:** 10.1098/rspb.2024.2485

**Published:** 2025-07-02

**Authors:** Stacy Rosenbaum, Anup Malani, Amanda J. Lea, Jenny Tung, Susan C. Alberts, Elizabeth A. Archie

**Affiliations:** ^1^ Department of Anthropology, University of Michigan, Ann Arbor, MI, USA; ^2^ University of Chicago Law School, University of Chicago, Chicago, IL, USA; ^3^ Department of Biological Sciences, Vanderbilt University, Nashville, TN, USA; ^4^ Primate Behavior and Evolution, Max-Planck-Institute for Evolutionary Anthropology, Leipzig, Sachsen, Germany; ^5^ Evolutionary Anthropology and Biology, Duke University, Durham, NC, USA; ^6^ Biological Sciences, University of Notre Dame, Notre Dame, IN, USA

**Keywords:** developmental plasticity, silver spoon hypothesis, predictive adaptive response, fitness, developmental adaptive response

## Abstract

In evolutionary ecology, two classes of explanations are frequently invoked to explain early life effects on adult outcomes. Developmental constraints (DC) explanations contend that the costs of early adversity arise from limitations adversity places on optimal development. Adaptive response (AR) hypotheses propose that later life outcomes will be worse when early and adult environments are poorly ‘matched’. Here, we use recently proposed mathematical definitions for these hypotheses and a quadratic-regression based approach to test the long-term consequences of variation in developmental environments on fertility in wild baboons. We evaluate whether low rainfall and/or dominance rank during development predict three female fertility measures in adulthood, and whether any observed relationships are consistent with DC and/or AR. Neither rainfall during development nor the difference between rainfall in development and adulthood predicted any fertility measures. Females who were low-ranking during development had an elevated risk of losing infants later in life, and greater change in rank between development and adulthood predicted greater risk of infant loss. However, both effects were statistically marginal and consistent with alternative explanations, including adult environmental quality effects. Consequently, our data do not provide compelling support for either of these common explanations for the evolution of early life effects.

## 1. Introduction

In many species, including humans, exposure to socioecological adversity early in life predicts negative outcomes in adulthood, including poor health, reduced fitness, compromised social functioning and shorter lifespans [1–7]. Given the diverse range of species in which such early life effects are observed, there is considerable interest in understanding the evolutionary origins of the connections between early life experiences and adult outcomes [8–12].

Two major classes of conceptual models are commonly invoked to explain early life effects in the evolutionary ecology literature [13,14] and have influenced thinking about the evolution of early life effects in humans [9,11,15]. The first class, developmental constraints (also known as silver spoon) hypotheses, proposes that poor-quality early life environments lead to poor outcomes in adulthood, such as shortened lifespans or poor health [8,13,16]. This may occur due to morphological, physiological and/or behavioural tradeoffs that prioritize short-term survival but carry long-term costs [17]. The second class proposes that organisms adopt a phenotype suited to either some aspect of their predicted future (the predictive adaptive response hypothesis, including both ‘internal’ and ‘external’ predictive adaptive responses), or to their developmental environment (the developmental adaptive response hypothesis) [17–19]. In either scenario, they incur fitness costs when the phenotype they adopt (e.g. a ‘fast’ life history strategy or a particular metabolic rate or body size) was not the best choice for the circumstances they face later in life, whether those circumstances are some feature of their external environment or some aspect of their own internal state. Importantly, the two classes of models do not have to be mutually exclusive [20–23]. Developmental constraints hypotheses generate predictions about the downstream effects of the quality of an organism’s early life environment, while adaptive response hypotheses generate predictions about the relationship between adult outcomes and environmental (or somatic) stability across the lifespan.

In animals with slow life histories, there is more empirical support for developmental constraints than for adaptive response hypotheses (reviewed in [12,14,24]). Many studies have found that organisms fare worse when they experience poor-quality developmental environments [6,25,26]. However, it is difficult to know which hypothesis (or hypotheses) best explain this relationship. The intertwined nature of the variables complicates empirical tests. Early life conditions and the difference between early life and adult conditions are not independent of one another, because one is used to calculate the other. Consequently, commonly applied tests to differentiate the hypotheses may be vulnerable to high error rates and conflate the two theories [27].

To help remedy this problem, we recently published formal (mathematical) definitions of developmental constraints, developmental adaptive response and predictive adaptive response hypotheses and proposed empirical tests derived from the definitions [27]. In brief, our definition of the developmental constraints model states that experiencing a worse developmental environment leads to worse outcomes in adulthood. Our definition of the developmental adaptive response model states that organisms alter their phenotype to adapt to their developmental environment, while our definition of the predictive adaptive response model states that organisms adapt their phenotype in anticipation of a predicted future. Both of these adaptive response definitions specifically posit that adult outcomes are a function of the difference between developmental and adult environments, which arise as a consequence of the organism’s ‘choice’ of a phenotype based on the cues they receive from the developmental environment. All three definitions are agnostic as to how, mechanistically, the connection between environmental features and adult outcomes occurs. More details can be found in §2d and in [27].

The developmental and predictive adaptive response hypotheses can be difficult to distinguish empirically [14,28]. One strategy for operationalizing prediction is to assume that organisms predict that some feature(s) of their developmental environment (e.g. the amount of rainfall they experience) will be similar in adulthood [11,15,29,30]. If this prediction is accurate (i.e. organisms experience little difference between their developmental and adult environments), then their outcomes will be better than if their prediction were incorrect (i.e. they experience large differences). However, the developmental adaptive response hypothesis makes the same prediction: organisms will fare worse if their adult environment is not suited to the phenotype they adopted in response to their developmental environment. Because the hypotheses cannot be distinguished using measures of environmental difference and adult outcomes alone [14,27–29], here we group them as a single adaptive response (AR) hypothesis.

We proposed our formal definitions and an analysis strategy in [27], but we did not apply them to real data. Here, we do so for the first time by evaluating the evidence for developmental constraints (DC) and AR in wild female baboons monitored by the Amboseli Baboon Research Project [31]. Baboons are excellent subjects for this research because they live in a highly variable environment that can generate considerable differences in the socioecological conditions experienced by the same animal throughout its life [4,31,32]. Additionally, this population has been studied for more than 50 years, so the types of data necessary to investigate the long-term effects of early life are available (e.g. [4,33–35]).

In social animals, aspects of both early life ecological and social environments may affect adult outcomes [36,37]. Here, we examine the effects of two key socioecological variables. The first is dominance rank, which predicts priority of access to resources that help determine fitness outcomes [7,38,39]. Female baboons have linear dominance hierarchies with a strong pattern of non-genetic matrilineal rank inheritance [40,41]. Thus, many female baboons hold a rank in adulthood that is similar to the rank their mother held when they themselves were born. However, due to group fissions and matriline overthrows (where one family successfully challenges a higher-ranked family, leading to position changes in the dominance hierarchy), some animals end up higher or lower-ranking than the rank they experienced (via their mother) during development [31,41]. The other variable is rainfall, which predicts food availability and thus female fertility [33,42,43]. Due to Amboseli’s semi-arid and seasonal ecosystem with more than fourfold variance in annual rainfall [44,45], during adulthood a female might experience years in which rainfall was similar to what she experienced during her first year of life, and years in which it was very different.

We capitalize on these characteristics of baboon socioecology to test the DC and AR hypotheses. Specifically, we test the DC hypothesis by asking whether low rainfall or low-rank during development predicts lower odds of conceiving, giving birth to a live infant, and/or raising an infant to weaning age as an adult. Next, we test the AR hypothesis by asking whether larger dominance rank or rainfall differences between development and adulthood predict these same three outcomes. Conceptions, live births and infant survival are the components of fertility that determine lifetime reproductive success and are particularly useful for our tests for three reasons. First, because they occur on a relatively short timescale, we can operationalize the 'match’ between developmental and adult environments. Second, when testing the AR hypothesis, using fertility components rather than a single lifetime reproductive success measure allows us to compare the same female to herself at different points in time. This is a stronger test of the AR theory than relying on across-animal comparisons due to the many inevitable differences between individual animals that we cannot control for. This would not be possible if each female had only a single value in the data set (see details in §2d). Finally, this strategy maximizes our sample size, because we can include females whose reproductive lifespans are still in progress. Our data enable us to investigate the relative evidence for DC and AR in one of the largest samples available for wild social mammals, drawing on a theory-aligned statistical method to evaluate the effects of developmental environments and developmental/adult environment differences on key fertility measures.

## 2. Methods

### (a) Study subjects

Our subjects were 295 wild female baboons that reside in the Amboseli ecosystem in southern Kenya. Baboons in this population have primarily yellow baboon (*Papio cynocephalus*) ancestry, but also near-universal minority ancestry from Anubis baboons [46,47]. They have been studied since 1971 by the Amboseli Baboon Research Project (ABRP) [31], which collects longitudinal demographic, ecological and life history data on individually recognized animals. Reproductive state (e.g. cycling, pregnant), the timing of events (e.g. births, conceptions) and females’ ages were known based on direct, nearly daily observations of females done by experienced ABRP researchers. The data in our analyses span the years of 1974 to 2023.

### (b) Outcome variables

Our outcomes were three fertility measures. The first was whether a female **conceived in a given observation month (y/n**), conditional upon having been cycling on the first day of the month (i.e. not pregnant or in postpartum amenorrhea). The second outcome was whether or not a female **gave birth to a live infant (y/n**), conditional upon being pregnant (note that we miss pregnancies that end in early term miscarriages [48]). The third outcome was whether a female **successfully raised an infant to 70 weeks of age (y/n**) (the average age at weaning [49]), conditional upon having given birth to a live infant. Sample sizes in the rainfall models (table 1) are slightly smaller than sample sizes in the rank models (table 2) because rainfall data collection started in 1976 and rank data in 1974. Pregnancy or infant survival data that were censored (infants who had not yet reached 70 weeks, or in-progress pregnancies) were dropped from the analyses. For the infant survival analysis, we also excluded observations where the subject died before her infant, as early maternal loss strongly predicts subsequent infant death.

**Table 1.** Basic descriptive information for rainfall analyses.

model	unit of analysis	outcome (y/n)	adult rainfall measure
conceptions (*n* = 1154)	cycling-months (*n* = 8404; 294 females)	conceived in month	monthly mean in year before cycling month
live births (*n* = 1065)	pregnancies (*n* = 1250; 257 females)	gave birth to live infant	monthly mean in year before end of pregnancy
infant survival (*n* = 686)	live births (*n* = 929; 236 females)	infant survived to 70 weeks	monthly mean in year before infant dies or reaches 70 weeks

**Table 2.** Basic descriptive information for dominance rank analyses.

model	unit of analysis	outcome (y/n)	adult rank measure
conceptions (*n* = 1158)	cycling-months (*n* = 8,433; 295 females)	conceived in month	mean in 3-month window around cycling month
live births (*n* = 1069)	pregnancies (*n* = 1,254; 258 females)	gave birth to live infant	mean over duration of pregnancy
infant survival (*n* = 688)	live births (*n* = 933; 237 females)	infant survived to 70 weeks	monthly mean in year before infant dies or reaches 70 weeks

### (c) Predictor variables

**Figure 1.**
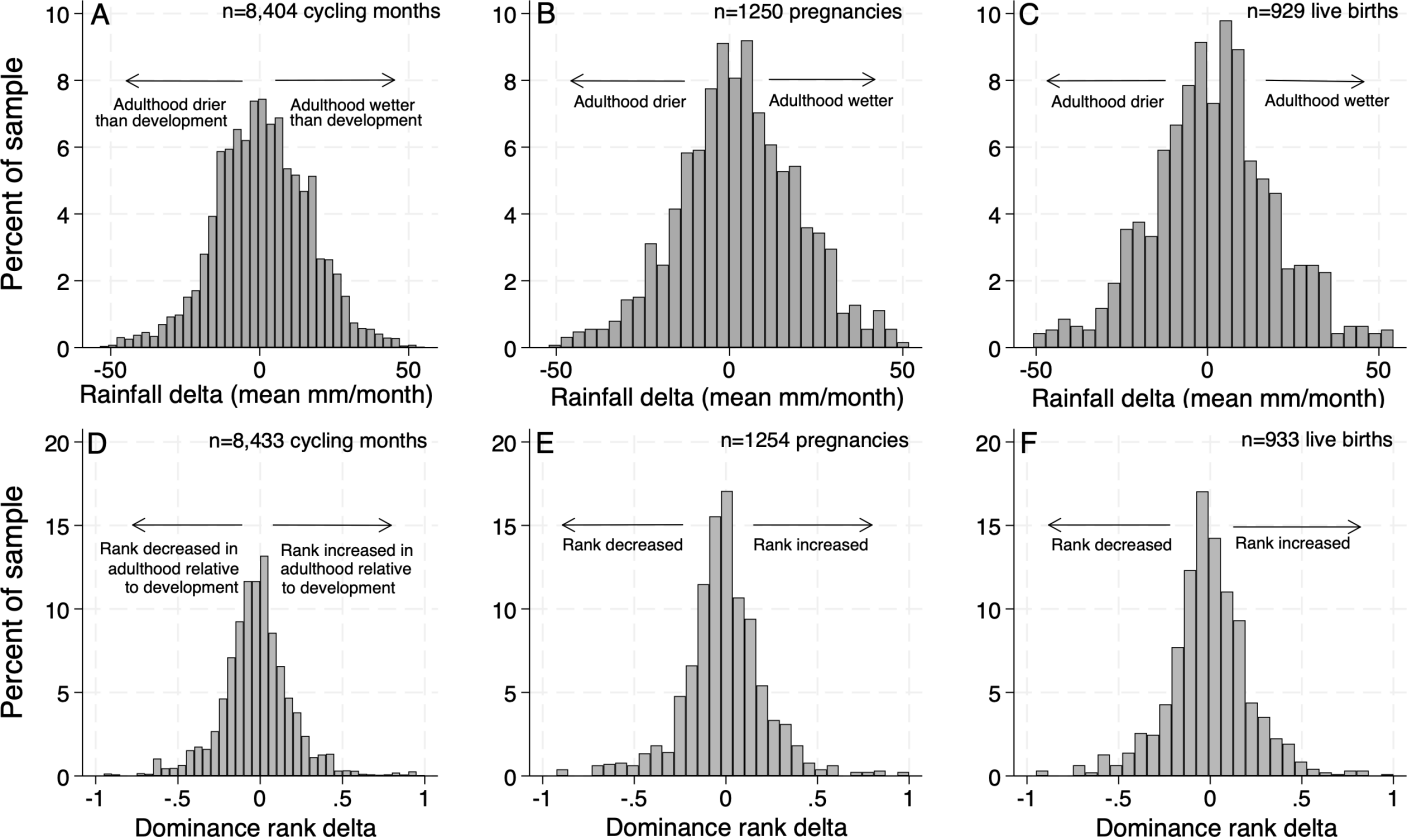
Difference between rainfall (top row) and dominance rank (bottom row) between development and adulthood. **Top row:** The distribution of rainfall deltas (i.e. the difference between the amount it rained during subjects’ first year of life and the amount it rained in the year of adulthood in which the outcome was measured (details in table 1)), expressed in mean mm per month. Zero on the *x*-axis indicates that it rained the same amount during development as it did during the year the fertility outcome was measured. **Bottom row:** The distribution of dominance rank deltas (i.e. the difference between subjects’ mothers’ proportional dominance rank when the subject was born, and the subjects’ proportional dominance rank in the period of adulthood in which the outcome was measured (details in table 2)). Zero on the *x*-axis represents a perfect match between rank at birth and adult rank when the outcome was measured.

**Rainfall.** To test whether female baboons have better reproductive outcomes if they (i) are born in higher-rainfall years, and (ii) experience smaller rainfall deltas (i.e. the rainfall they experienced in adulthood more closely matches the rainfall they experienced during development), we used daily precipitation data collected from a rain gauge at the ABRP field camp. For rainfall during development, we calculated average monthly rainfall in the subject’s first year of life. We used 12 month windows for rainfall because the Amboseli ecosystem is highly seasonal [45]; with shorter windows, rainfall could vary considerably simply due to the months in which the window happened to occur (e.g. August–December would not be comparable to February–June because of normal seasonal rainfall patterns). Averaged across data sets (table 1), mean monthly rainfall during development was 28.80 mm per month (s.d. = 0.31, range = 7.43–63.92). For rainfall in adulthood, we calculated average monthly rainfall in the 12 months before the fertility event in question. Averaged across the three data sets (table 1), mean monthly rainfall during adulthood was 30.53 mm per month (s.d. = 0.98, range = 7.92–66.76). To calculate rainfall deltas, we subtracted rainfall during development from rainfall in adulthood. Histograms of the difference between rainfall during development and adulthood can be found in the top row of figure 1 (conception: panel A; live birth: panel B; and infant survival: panel C). Detailed statistics are available in section B of the electronic supplementary material.

**Dominance rank.** To test whether female baboons have better reproductive outcomes if (i) they are higher-ranking during development, or (ii) they have smaller rank deltas (i.e. their dominance rank in adulthood is similar to their rank during development), we used animals’ proportional dominance ranks. These represent the proportion of adult female group members that the subject outranks [50,51] (e.g. 0.9 means that the female outranks 90% of the adult females in her group). Dominance was determined by the outcomes of all observed, decided agonistic interactions between females and was estimated monthly [50,51]. Further rank details can be found in section C of the electronic supplementary material and in [51].

Rank during development was defined as the average rank of the subject’s mother over the three-month span centring on the subject’s birth (i.e. the month of the birth, plus the month before and the month after). Rank in adulthood was calculated as described in table 2. Adult rank measures were aggregated over time windows we felt were of biological relevance for the outcome in question (e.g. for a short-term event like conception, rank over the prior year may be less biologically relevant, but could be more so if the outcome is a sustained ‘event’ like pregnancy or an infant’s life). For rank during development and during potential conception months in adulthood, using the mean of a three-month window allowed us to retain more observations than using only the observation month would, due to occasional data gaps. The correlation between average rank in the three-month windows and rank strictly during the month of interest was >0.99. Rank position is relatively stable for female baboons, so calculating rank over different periods is unlikely to change rank values or results. Histograms of the difference between rank during development and adulthood can be found in the bottom row of figure 1 (conception: panel D; live birth: panel E; and infant survival: panel F).

#### (i) Covariates

Age and group size can both influence female fertility, so we included these as covariates in our models [33,38,42]. We used age at the start of the conception month, at pregnancy termination, and at infant birth, as appropriate for a given model (tables 1 and 2). We also included a squared age term because old and very young females tend to be less fertile [33,42,52]. In the electronic supplementary material (section D, tables A8–A13), we also present versions of the models that interact the environmental variables with age indicators, allowing us to test the DC and AR hypotheses over different age segments (e.g. [53]). The youngest (<6 years old) and oldest (20+ years old) animals add noise to the data, but the results are qualitatively similar to those presented in the main text. Group size was defined as the average number of group members each day during the cycling month, across the pregnancy and across the resulting infant’s life, as relevant for a given model. See electronic supplementary material, section D for additional covariate details.

Females who have not yet given birth to a live infant have lower odds of conceiving relative to other females, and females whose last infant died before weaning require fewer cycling months to conceive than females whose prior infant survived [38,54,55]. We did not include parity or prior infant status in the conception models because we are unsure if our predictors of interest influence time until first conception or time until conception after an infant loss. For example, if a female who experienced drought during development is more likely to lose her infant than females who did not experience drought, controlling for infant loss in conception models could mask the relationship between drought during development and adult conception probability.

### (d) Analysis strategy

Our formal versions of the DC and AR hypotheses posit that variation in the environment (either early life itself or early life/adult environment deltas) is causally responsible for differences in later-life outcomes. The ideal test of the DC hypothesis would be to vary the animals’ early life environment while holding all else constant. Since this is impossible in natural populations, we compare across individuals under the assumption that rank and rainfall are independently distributed across our study subjects [56,57]. For rainfall, this is probably a reasonable assumption, as no property of the baboons themselves influences how much it rains. For rank, this is questionable (see discussion in §4). Females of different ranks are likely different from one another in other, unobserved ways.

In contrast to DC, where only across-individual comparisons are possible, for the AR hypothesis we can make both across- and within-individual comparisons. This is because the same female can be observed multiple times in adulthood: a female baboon could experience drought in one year but plentiful rainfall in another, or change rank between years.

#### (i) Definitions and tests

Our regression models and their associated tests are derived from the mathematical definitions of DC and AR provided in table 3. While we provide a brief overview below, full details are available in [27].

**Table 3.** Formal definitions and derived prediction of theories for the relationship between the quality of developmental environments and adult outcomes. Notes: $y_{0}$ = outcome measured during development; $y_{1}$ = outcome measured during adulthood; $e_{0}$ = developmental environment; $e_{1}$ = adult environment; $E(e_{1})$ = adult environment the organism expects; Δe = difference between developmental and adult environments; *p* = phenotypic adaptation adopted in response to $e_{0}$ or $E(e_{1})$. Without loss of generality, definitions assume that functions relating outcomes to environments are continuously differentiable. For details on these definitions and a discussion of their extension to categorical variables, see [27].

theory	definition	observable variation	prediction
developmental constraints	$\frac{\partial y_{1}}{\partial e_{0}}>0$	$\left(y_{1},e_{0}\right)$	$\frac{\partial y_{1}}{\partial e_{0}}>0$
adaptive responses			
(a) predictive	$E(e_{1})=e_{0};\frac{\partial p}{\partial E(e_{1})}<0;\frac{\partial^{2}y_{1}}{\partial p\partial e_{1}}<0$	$\left(y_{1},\Delta e,p\right)$	$\frac{\partial y_{1}}{\partial |\Delta e|}<0$
(b) developmental	$\frac{\partial p}{\partial e_{0}}<0;\frac{\partial^{2}y_{0}}{\partial p\partial e_{0}}<0$	$\left(y_{1},\Delta e,p\right)$	$\frac{\partial y_{1}}{\partial |\Delta e|}<0$

DC proposes that if an organism experiences a low-quality developmental environment, it will exhibit poor adult outcomes. This can be represented as:


(2.1) $$\begin{array}{lrr} & \;y_{1}=f(e_{0})\text{ such that }\frac{\partial y_{1}}{\partial e_{0}}>0, & \end{array}$$

where $y_{1}$ is adult outcomes (here, a fertility event), f is an increasing function, and $e_{0}$ is the developmental environment (see the first row of table 3). Higher $y_{1}$ and $e_{0}$ indicate better outcomes and higher-quality environments, respectively. Without loss of generality, we assume that functions relating outcomes to environments are continuously differentiable. We use a partial derivative (∂) because we are holding all else constant.

AR’s causal chain proposes that organisms experience something in their developmental environment; develop an phenotype that is optimal given that developmental experience; and then exhibit better outcomes in adulthood if they chose a phenotype suitable to that experience. In our use case, this is how much rainfall and what dominance rank the baboon experiences; hereafter, we will refer to these together as environment.

The causal chain is indifferent to whether the organism ‘chose’ the phenotype because it predicted that its adult environment would be similar to its developmental environment (see row (a) in table 3), or whether it chose the phenotype to improve its outcomes during development and it just so happens that its adult environment is similar to what it experienced during development (see row (b) in table 3).^1^

This hypothesis implies the following definition:


(2.2) $$\begin{array}{lrr} & \;y_{1}=g(e_{1}-e_{0}),\;\text{where}\;\frac{\partial y_{1}}{\partial |e_{1}-e_{0}|}=\frac{\partial y_{1}}{\partial |\Delta e|}<0, & \end{array}$$

where Δe is the difference between the environment in adulthood and the environment during development. In this case g is a decreasing function because outcomes will get worse as |Δe| gets bigger. Again, we use ∂ because we are holding all else constant.

Since outcomes could be affected both by $e_{0}$ (equation (2.1)) and by Δe (equation (2.2)), we propose that adult outcomes can be described by a very general mathematical function $y_{1}=F(e_{0},|\Delta e|)$*,* where F is a continuous differentiable function (i.e. a curve) over developmental and adult environments. A simple regression equation of the form $y_{1}=\beta +\beta_{0}e_{0}+\beta_{d}|\Delta e|+u$ is a linear approximation of F, but this linear model is probably too simple to capture real-world dynamics. For example, better developmental environments might have diminishing benefits or larger environmental deltas might have diminishing negative effects on adult outcomes.

To accommodate this, we use a second-order Taylor expansion of F around $e_{0}=e_{1}=|\Delta e|=0$, i.e. a sum of first and second powers of the inputs to F. Taylor approximations are a standard way to develop regression models and prescribe that all polynomials of a chosen order are included in the regression [58]. This suggests a quadratic regression model of the form:


(2.3) $$\begin{array}{lrr} & \;y_{1}=\gamma +\gamma_{0}e_{0}+\gamma_{d}|\Delta e|+\gamma_{00}{e_{0}}^{2}+\gamma_{\text{dd}}|\Delta e{|}^{2}+\gamma_{0d}e_{0}|\Delta e|+u, & \end{array}$$

where the subscript 0 on γ indicates the coefficient is on $e_{0}$, d indicates it is on |Δe|, 0d indicates it is on $e_{0}|\Delta e|$, and 00 indicates that it is on ${e_{0}}^{2}$. This regression is useful due to its flexibility. It can accommodate a range of patterns in the data, and results using simulated data indicate that it yields 100% sensitivity for DC tests and 90% sensitivity for AR tests (both described below) under a wide array of parameter values [27].

The above regression (equation (2.3)) implies different statistical tests for DC and AR. We test for DC using the following inequality:


(2.4) $$\begin{array}{lrr} & \frac{\partial y_{1}}{\partial e_{0}}=\gamma_{0}+{2\gamma }_{00}e_{0}+\gamma_{0d}|\Delta e|>0. & \end{array}$$

This says that if the partial derivative of adult outcomes ($y_{1}$) with respect to the quality of the developmental environment ($e_{0}$) is positive (i.e. adult outcomes improve as a function of better developmental environments), then we can reject the null hypothesis that there is no relationship between developmental environment and adult outcomes.

To test for AR, we use the following inequality:


(2.5) $$\begin{array}{lrr} & \frac{\partial y_{1}}{\partial |\Delta e|}=\gamma_{d}+2\gamma_{\text{dd}}|\Delta e|+\gamma_{0d}e_{0}<0 & \end{array}$$

This says that if the partial derivative of adult outcomes with respect to the size of the *difference* between developmental and adult environments (|Δe|) is negative, then we can reject the null that there is no relationship between the degree of environmental mismatch and adult outcomes. Further details on the theoretical motivation for the quadratic regression approach and the derivation of the hypothesis tests are available in [27]. To assist other authors with the implementation of quadratic models and derivative tests, a link to R and Stata code can be found in the data availability statement.

Following [27], a term for the quality of the adult environment is not included due to the non-independence of $e_{0}$, Δe and $e_{1}$. If $e_{0}$ is held constant and $e_{1}$ is different than $e_{0}$, then it is impossible to tell if any observed changes in the outcome are due to the value of $e_{1}$ or to Δe. Following [27], we assume that what best represents the biological phenomenon under consideration is that $e_{0}$ and Δe are independent variables that collectively generate $e_{1}$ via the relationship $e_{0}$ + Δe = $e_{1}$. We make this assumption because an organism does not experience $e_{1}$ independent of what it experienced during $e_{0}$. Because of this assumption [59], $e_{1}$ is simply an endogenous byproduct of the structure of the theoretical model, and thus not a testable variable.

Our models cluster on individual baboon IDs. We present the results of models with different fixed effects specifications. The first is a social group fixed effect, which compares across individuals within social groups (as opposed to a model where social group was treated as a random effect, which would compare across social groups [60]). This strategy helps account for differences in habitat quality and other factors that may vary from group to group. For example, if one group had an exceptionally high-quality home range and another an exceptionally poor-quality one, then in theory low-ranking (or drought-experiencing) females from the prior group might always outperform females from the later group. If this were the case, comparing across all females, instead of comparing females within social groups, could generate misleading results.

For our tests of AR, we also present results from models with animal identity as a fixed effect. This means that the models are comparing females to themselves at different points in time. Using identity as a fixed effect rather than a random effect means we can compare how changing levels of environmental mismatch are associated with fertility outcomes for the same individual [60,61]. This is both theoretically consistent with our mathematical definitions of DC and AR, and is the strongest test of the AR hypothesis, since it eliminates other sources of between-animal variation. While the group fixed effect model described above is thus not the preferred choice for testing AR, we present the group fixed effects results for AR in the interest of conducting one set of tests where the DC and AR hypotheses are structured the same way (i.e. in both cases, the comparison being made is across females but within groups). We cannot test DC when including the individual-level fixed effect because each individual only experiences one developmental environment.

The structure of the analyses (two hypotheses and two environmental variables, with two fixed-effects specifications for each environmental variable) means we conduct multiple hypothesis tests. We therefore also calculated sharpened two-stage q-values that take into account the observed distribution of p-values across tests using a false discovery rate approach [62,63]. These q-values are only reported in the text for findings where the uncorrected *p*-value was ≤0.100.

## 3. Results

In the main text, we provide variable summaries and results of the derivative tests associated with each model in table 4. Coefficients and *p*-values for every term for all models can be found in electronic supplementary material, section A.

**Table 4.** Results from quadratic models examining the effect of developmental environment and developmental/adult environment deltas on different fitness outcomes.

(a)	outcome: probability of conception			
environmental variable	rank (*n* = 8433)		rain (*n* = 8404)	
fixed effect specification	group	individual	group	individual
	*test of theories: marginal effects and p-values*			
DC: $\partial y/\partial e_{0}$	0.009		> −0.001	
	(0.540)		(0.835)	
AR: ∂y/∂|Δ|	−0.021	0.020	< 0.001	< 0.001
	(0.573)	(0.749)	(0.643)	(0.461)
	*context for marginal effects: sample means and sd*			
y	0.137		0.137	
	0.344		0.344	
e0	0.537		28.457	
	0.285		10.510	
|Δ|	0.158		12.541	
	0.155		9.822	

(b)	outcome: giving birth to a live infant			
environmental variable	rank (*n* = 1254)		rain (*n* = 1250)	
fixed effect specification	group	individual	group	individual
	*test of theories: marginal effects and p-value*			
DC: $\partial y/\partial e_{0}$	0.007		0.001	
	(0.849)		(0.524)	
AR: ∂y/∂|Δ|	0.110	−0.103	0.001	0.001
	(0.272)	(0.551)	(0.394)	(0.582)
	*context for marginal effects: sample means and sd*			
y	0.852		0.852	
	0.355		0.355	
e0	0.533		28.885	
	0.299		11.063	
|Δ|	0.161		13.749	
	0.162		10.879	

(c)	outcome: successfully raising an infant to 70 weeks			
environmental variable	rank (*n* = 933)		rain (*n* = 929)	
fixed effect specification	group	individual	group	individual
	*test of theories: marginal effects and p-value*			
DC: $\partial y/\partial e_{0}$	0.108		> −0.001	
	(0.043)		(0.838)	
AR: ∂y/∂|Δ|	−0.204	−0.485	0.002	0.001
	(0.153)	(0.081)	(0.354)	(0.451)
	*context for marginal effects: sample means and sd*			
y	0.737		0.737	
	0.440		0.440	
e0	0.529		29.049	
	0.299		11.217	
|Δ|	0.167		14.066	
	0.161		11.347	

### (a) Tests of the developmental constraints hypothesis

#### (i) The effects of rainfall during development

None of our models provided any evidence that lower rainfall during development predicted worse fertility outcomes in adulthood. The amount of rainfall during development did not predict the likelihood of conceiving (contingent upon being cycling, p=0.835; table 4a), giving birth to a live infant (contingent on pregnancy, p=0.524; table 4b), or successfully raising an infant to 70 weeks (contingent on having given birth to a live infant, p=0.838; table 4c). Full model details can be found in electronic supplementary material, tables A.1, A.2 and A.3.

#### (ii) The effects of rank during development

Females who were lower-ranking during development were no less likely than their peers to conceive (p=0.540; table 4a) or to give birth to a live infant (p=0.849; table 4b). Full model details are available in electronic supplementary material, tables A.1 and A.2.

Consistent with the DC hypothesis, females born to lower-ranking mothers were somewhat less likely to successfully raise an infant to 70 weeks than their peers who were born to higher-ranking mothers (p=0.043; table 4c, with full details in electronic supplementary material, table A.3). Females whose own mothers were in the 10th percentile of rank when they were born were 13.18% more likely to have their infant die before weaning than females whose mothers are in the 90th percentile of rank when they were born (s.d. = 22.08%). Given that the mean infant survival probability was 73.74%, this translates to a 9.72% difference in the overall odds of infant survival for females in the 10th versus 90th rank percentiles (i.e. 13.18% of 73.74% is 9.72), or the equivalent of 25.55% of one standard deviation. After correcting for multiple testing, the q-value for this result was q=0.095.

### (b) Tests of the adaptive response hypothesis

#### (i) The effects of rain deltas

Females were not less likely to conceive (p=0.461; table 4a), give birth to a live infant (p=0.582; table 4b), or successfully raise an infant to 70 weeks (p=0.451; table 4c) when they experienced a large rainfall delta than when they experienced a small rainfall delta. The effects were qualitatively similar when comparing across females, within groups. In the group fixed effect models, for all three outcome variables, p>0.353 (table 4a–c). Full model details are available in electronic supplementary material, tables A.1, A.2 and A.3.

#### (ii) The effects of rank deltas

Females were not less likely to conceive (p=0.749; table 4a) or to give birth to a live infant (p=0.551; table 4b) when their dominance rank deltas were bigger than when they were smaller. This was qualitatively similar when comparing across females, within groups (p>0.271 for both outcome variables; see table 4a,b). Full details are in electronic supplementary material, tables A.1 and A.2.

We identified no statistically significant patterns that supported the AR hypotheses, though there was one case in which results approached consistency with the mismatch prediction of AR. When females had larger dominance rank deltas, they were more likely to lose an infant before weaning than when they had smaller rank deltas (where p=0.081; table 4c). Based on a mean infant survival probability of 73.74%, when a female was in the 90th percentile of rank difference she was 59.19% more likely to lose an infant before weaning than when she was in the 10th percentile of rank difference. This translates to a 43.65% difference in the overall odds of infant survival between the 10th and 90th percentiles of rank delta.

**Figure 2.**
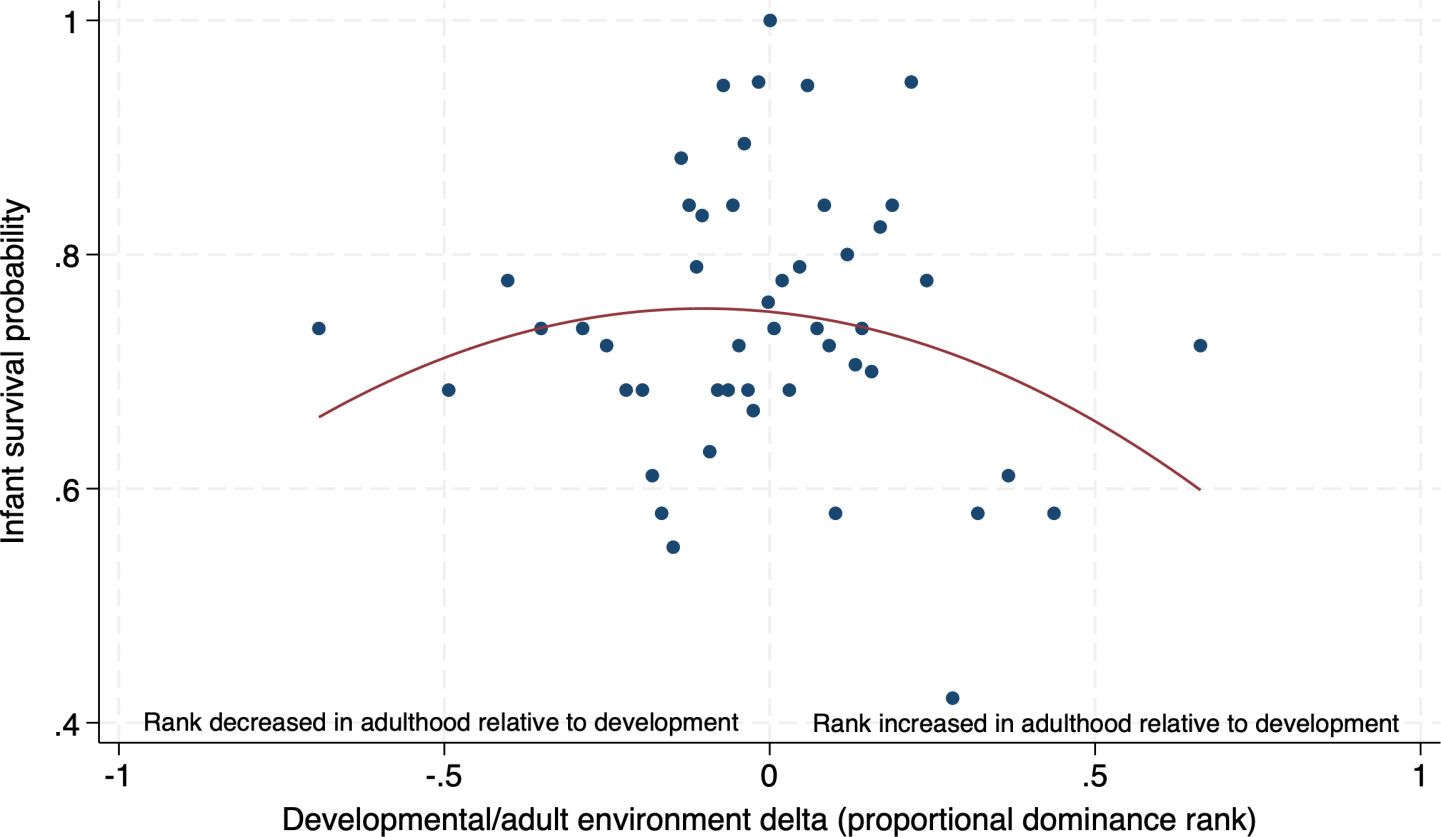
The relationship between dominance rank deltas and infant survival probability. We observed non-statistically significant support for the adaptive response (AR) hypothesis; females had a lower chance of successfully raising an infant to the average age at weaning when there were greater differences between their rank during development and their rank in adulthood than they did when these differences were smaller (unadjusted p=0.081, see table 4c; after multiple testing adjustment, q=0.441). The centre of the *x* axis (0) represents a developmental/adult environment delta of zero, meaning that the female held the same rank in adulthood as she did during development. The plot shows raw (i.e. unadjusted) data grouped into 50 bins, overlaid with a quadratic fit line. Total sample size = 933 live births, so each bin contains approximately 19 data points (2% of the sample).

Figure 2 depicts a quadratic fit line over 50 bins of the raw data (i.e. each displayed point is an average of the observations of about 2% of the sample). While figure 2 visually suggests that the magnitude of positive changes in rank (the right-hand side of the plot) may be slightly larger than the magnitude of negative changes in rank (the left-hand side of the plot), these differences are not statistically significant (p>0.250; see electronic supplementary material, section E for details on how these analyses were performed). Hence, the pattern is being driven both by females who had lower ranks in adulthood compared to development, and higher adult ranks compared to development.

Though the size of the effect of rank deltas on infant survival is large, we caution that the uncertainty in the effect size estimate is also high, with a sharpened *q*-value well above statistical significance thresholds after a multiple testing adjustment (q=0.441). The results were qualitatively similar, though with smaller effect size estimates, when the comparison was across females, within groups (42% of the within-individual effect size, p=0.153; table 4c). Full details of these infant survival models can be found in electronic supplementary material, table A.3.

## 4. Discussion

We find that low early-life rainfall and low early life rank—two important aspects of developmental environments for wild female baboons [4,33,39,43]—do not play a substantive role in female fertility outcomes for wild baboons. Being born during low-rainfall years or to a low-ranking mother did not significantly predict adult fertility parameters in our models, with one possible exception: females born to low-ranking mothers were somewhat more likely to have an infant die before weaning than females born to high-ranking mothers were. However, this result is not statistically well-supported, and our sample size is large compared to most wild primate studies.

Our results, therefore, do not provide clear evidence in favour of either DC or AR for the environmental variables and outcomes we considered here. Prior Amboseli results [33] concluded that early life drought predicted reduced probabilities of conception and resumption of cycling following post-partum amenorrhea, specifically when females experienced drought conditions in adulthood [33]. These results were interpreted as support for DC because females born during drought did worse when drought recurred in adulthood than they did when their adult environments were more favourable. The dataset for the earlier analysis was constructed differently: [33] binarized (drought/no drought) early life environments and asked whether fertility outcomes differed for females that later lived through both a normal rainfall year and drought in adulthood (*n* = 50 females fit these criteria). Some differences in findings are thus probably related to differences in the composition and size of the datasets. Others are probably an outgrowth of analysis strategy: for example [33], controlled for reproductive state rather than conditioning females’ inclusion on reproductive state. Furthermore, the earlier analysis relied on a model containing an interaction term between the developmental and adult environments, instead of using a quadratic regression and evaluating the relevant partial derivatives. This analysis strategy increases the reliability and interpretability of DC and AR tests since it results in markedly better sensitivity and specificity than relying on a regression that contains a developmental environment/adult environment interaction [27].

Our findings emphasize the importance of methodological choices when testing these hypotheses, which may be one of several potential reasons why the literature is still unclear on their relative importance in humans and other animals. While prior literature has generally found more support for DC than for AR (reviewed in [12,14,24,26]), there is considerable heterogeneity across the literature. For example, in support of DC, better early life environments are associated with greater lifetime reproductive success in bighorn sheep, Svalbard reindeer and spotted hyenas [22,64,65]. Meanwhile, three recent studies have not found clear evidence for decreased fitness due to some kinds of early life adversity. African elephants who lose their herd matriarch (not their mother) early in life do not appear to suffer fitness costs, nor do female mountain gorillas who lose their mother at a relatively young age [66,67]. Furthermore, drought in early life is not associated with reduced fitness in female elephants or banded mongooses [67,68]. Mammalian studies supporting the mismatch prediction of AR are scarce, although some have found evidence of AR in the form of phenotypes that develop as a result of exposure to certain environments *in utero* or in very early life [69,70].

One of the challenges in resolving AR and DC explanations is fundamental to any strictly observational research: we cannot vary only the predictors of interest while holding all else constant. Consequently, researchers must assume that the predictor of interest is randomly distributed across study subjects. In our study, this is a safe assumption in the case of rainfall, but less clear in the case of dominance rank. Females who hold different ranks during development are probably different from one another in various ways that affect later life fertility, such as growth rates, adult size or resource access [39,55,71]. And because female baboons non-genetically inherit their rank from their mothers (e.g. in our infant survival data set, the correlation between e0 and e1 is 0.71) [40,41], rank-related DC may simply arise from an effect of rank in adulthood. These intrinsic design issues complicate the interpretation of results, making experimental work on this subject especially valuable [70,72].

For the AR hypothesis, our within-individual comparisons somewhat mitigate the problem of confounding between-animal differences. However, even here, the experiences and environments of female baboons might be different when their rank deltas are large versus when they are small. For example, rising in rank is disproportionately associated with group fissions. Fissions primarily occur due to resource stress associated with large group size [73,74] and disrupt females’ lives, even if they result in status increase. Meanwhile, falling in rank may occur due to sickness or injury, which lower-ranking females can exploit. Consequently, multiple pathways can generate environmental mismatch effects, some of which can independently generate poor outcomes without requiring a causal effect of phenotypic choices organisms make. For example, in wild roe deer, increased viability selection in poor-quality environments may generate apparent fitness benefits to females who experienced matched poor-quality developmental and adult environments: because high-quality females were more likely to survive a harsh early environment, they performed better than the (on average) lower-quality females born in favourable environments, when both groups encountered harsh conditions later in life [75]. Most studies of human populations have ignored the potential role of viability selection and other alternative explanations that may generate a connection between environmental mismatch and adult outcomes (e.g. [29]).

Finally, the relative lack of evidence we find for effects of early life adversity on fertility measures suggests that shortened lifespans are probably the primary mechanism by which early adversity might decrease lifetime fitness. Prior analyses have demonstrated that experiencing more sources of early life adversity, including drought and being born to a low-ranking mother, leads to markedly shorter lifespans in this baboon population [4,35,76]. Lifespan is by far the single biggest contributor to females’ lifetime reproductive success, explaining 80–90% of the observed variation in Amboseli baboons [55,76]. Indeed, across taxa, shortened lifespans appear to be a common consequence of early life adversity, accounting for a significant proportion of the studies that provide evidence for DC (e.g. red squirrels [72]; chimpanzees [77]; Asian elephants [78]; hyenas [79]; reviewed in [25,37]). Consequently, for long-lived species like primates, lifespan analyses will probably be crucial to understanding the extent to which early life adversity compromises fitness.

In sum, our data do not provide clear support for either the DC or AR hypothesis as compelling explanations for differences in fertility outcomes in wild female baboons, at least when social status and rainfall are the environmental variables of interest. We hope that these analyses, which are structured to help avoid empirical conflation of the hypotheses, will motivate additional evaluation of the evidence for DC and AR using the statistical methods demonstrated here.

## Data Availability

The data reported in this paper can be found at [80]. The code to replicate the analysis is available at https://github.com/anup-malani/rosenbaum_etal_2024_baboon_dc_ar_mismatch. To assist other authors with the implementation of quadratic models and the associated tests of early life environment and environmental delta derivatives, a link to R and Stata code for this purpose can be found at https://github.com/anup-malani/PAR. Supplementary material is available online [81].

## Footnotes

^1^
There are two ways to test AR. The approach we employ here is to test whether differences between adult and developmental environments adversely affects adult outcomes (a ‘mismatch test’). Another approach is to test for the existence of adaptive phenotypic changes (a ‘phenotype test’) [27].
